# Nanotechnology in Ovarian Cancer: Advances in Early Diagnosis and Targeted Therapy to Enhance Patient Quality of Life

**DOI:** 10.3390/cells15141248

**Published:** 2026-07-10

**Authors:** Andreea Moise-Crintea, Tiberiu Vasile Ioan Nistor, Nadica Motofelea, Alexandru Catalin Motofelea, Liliana Ana Tuta, Minodora Manea

**Affiliations:** 1 Department of Molecular Sciences, University of Medicine and Pharmacy “Iuliu Hațieganu”, 400349 Cluj-Napoca, Romania; crintea.andreea@umfcluj.ro; 2 Department of Obstetrics and Gynecology, “Victor Babes” University of Medicine and Pharmacy, 300041 Timisoara, Romania; nadica.motofelea@umft.ro; 3 Department of Clinical Practical Skills, “Victor Babes” University of Medicine and Pharmacy, 300041 Timisoara, Romania; 4 Centre for Molecular Research in Nephrology and Vascular Disease/MOL-NEPHRO-VASC, “Victor Babes” University of Medicine and Pharmacy, 300041 Timisoara, Romania; alexandru.motofelea@umft.ro; 5 Nephrology Department, Faculty of Medicine, “Ovidius” University of Constanta, 900470 Constanta, Romania; tuta.liliana@univ-ovidius.ro; 6 Department of Neurosciences, Iuliu Hațieganu University of Medicine and Pharmacy, 400023 Cluj-Napoca, Romania; mmanea@umfcluj.ro

**Keywords:** nanotechnology, nanoparticles, ovarian cancer, early diagnosis, targeted drug delivery, drug resistance, personalized medicine, liposomes, gold nanoparticles, SPIONs, quality of life

## Abstract

Nanotechnology is rapidly advancing as a promising approach in ovarian cancer management, addressing key challenges such as late diagnosis, drug resistance, and systemic toxicity of conventional therapies. Nanoparticles—engineered at the 1–100 nm scale—possess unique physical and biological properties that make them well-suited for targeted drug delivery, imaging, and biomarker detection. In diagnostics, platforms such as gold nanoparticles, quantum dots, superparamagnetic iron oxide nanoparticles (SPIONs), and carbon-based nanomaterials have demonstrated the ability to improve sensitivity and specificity, enabling the detection of low-abundance biomarkers and enhancing imaging contrast. These advances could significantly improve the early-stage detection, where survival outcomes are most favorable. Therapeutically, nanoparticles offer controlled and sustained drug release, targeted delivery to specific tumor sites, and the ability to co-deliver multiple agents, including siRNA and mRNA, in order to overcome resistance pathways. Clinically, liposomal formulations such as Doxil, already demonstrate reduced toxicity and improved drug bioavailability, while polymeric, silica, gold, and magnetic nanoparticles continue to show encouraging results in preclinical and early clinical studies. Although challenges remain—including large-scale production, long-term safety evaluation, and regulatory complexity—the current body of evidence highlights nanotechnology’s transformative potential in ovarian cancer care. By enabling earlier detection, more precise targeting, and reduced systemic toxicity, nanomedicine represents a critical step toward improving both survival and quality of life in affected patients.

## 1. Introduction

Nanoparticles are defined as particles with sizes ranging from 1 to 100 nanometers, exhibiting unique physical, chemical, and biological properties that differentiate them from larger bulk materials. These properties arise from their nanoscale dimensions, which alter characteristics such as surface area, reactivity, and solubility [1,2]. Various substances can create nanoparticles, including metals such as gold and silver, as well as polymers, lipids, and ceramics. The versatility of nanoparticle shapes, including spheres, rods, and tubes, enables tailored adaptations for specific applications, particularly in drug delivery, imaging, and sensing technologies [3,4,5,6].

In the medical field, nanoparticles can be designed for targeted therapy, enhancing drug delivery to tumor sites while improving diagnostic techniques. Ovarian cancer remains one of the most formidable and lethal cancers affecting women globally, primarily due to late-stage diagnosis, resistance to conventional therapies, and a lack of effective early detection methods. Recent advancements in nanotechnology have opened new avenues for both diagnosis and treatment, providing hope for improved patient outcomes. The unique nanoscale properties make nanoparticles promising tools against ovarian cancer, revolutionizing its diagnosis, treatment, and follow-up [5,6,7,8].

Ovarian cancer is frequently diagnosed at advanced stages because of the absence of specific symptoms and the limited sensitivity of current early detection methods. Standard treatment strategies rely primarily on cytoreductive surgery followed by platinum- and taxane-based chemotherapy; however, therapeutic efficacy remains limited in advanced or recurrent diseases. In addition, many patients eventually develop chemoresistance through mechanisms such as altered drug transport, enhanced DNA repair capacity, and dysregulation of apoptotic pathways. Conventional chemotherapy is also associated with substantial systemic toxicity, including fatigue, nausea, myelosuppression, peripheral neuropathy, and increased susceptibility to infections, all of which negatively affect patient quality of life [7,8].

Furthermore, the ovarian tumor microenvironment (TME) plays a major role in disease progression and therapeutic resistance by promoting tumor survival, immune evasion, and reduced drug penetration. These limitations have stimulated growing interest in novel therapeutic strategies capable of improving tumor selectivity while minimizing off-target toxicity [9,10,11,12]. In this context, nanotechnology-based platforms have emerged as promising approaches for targeted drug delivery, controlled therapeutic release, multimodal imaging, and early biomarker detection. Such systems may enhance treatment efficacy, overcome multidrug resistance, and improve both survival outcomes and quality of life in ovarian cancer patients [13,14,15,16,17,18].

As ovarian cancer treatment evolves toward precision oncology, the integration of nanotechnology has the potential to reshape clinical management by simultaneously extending survival and preserving patient functionality and well-being. Liposomes and polymeric nanoparticles enhance drug bioavailability while minimizing systemic exposure, thereby reducing adverse effects that substantially impair quality of life [14,19,20,21]. Furthermore, nanoparticle-based delivery of siRNA and other molecular payloads enables tumor-specific gene modulation, contributing to improved efficacy and reduced off-target toxicity [3,22]. These developments highlight the transformative potential of nanotechnology in ovarian cancer care.

## 2. Nanoparticles for the Early Detection of Ovarian Cancer

**Figure 1:**
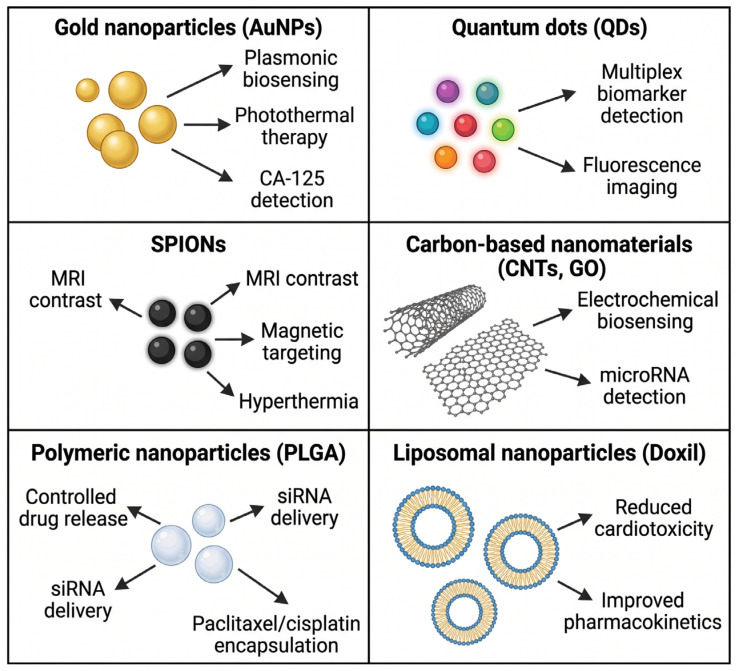
Overview of Nanoparticle Platforms Used in Ovarian Cancer Diagnosis and Therapy. Major classes of nanoparticles investigated in ovarian cancer, including gold nanoparticles (AuNPs), quantum dots (QDs), superparamagnetic iron oxide nanoparticles (SPIONs), carbon-based nanomaterials (CNTs, GO), polymeric nanoparticles (PLGA), and liposomal formulations (Doxil). Each platform exhibits distinct physicochemical properties that determine its diagnostic and therapeutic applications.

The rapid evolution of nanotechnology has generated a diverse spectrum of nanoscale platforms with applications that extend across early diagnosis, targeted therapy, and multimodal imaging in ovarian cancer [23]. Each nanomaterial class ranging from gold nanoparticles (AuNPs) and quantum dots (QDs) to superparamagnetic iron oxide nanoparticles (SPIONs), carbon-based nanostructures, polymeric carriers, liposomal formulations, and nucleic acid–based delivery systems exhibits distinct physicochemical properties that determine its biological behavior, therapeutic potential, and translational relevance. Understanding these differences is essential for evaluating their capacity to enhance biomarker detection, improve drug accumulation within tumors, overcome chemoresistance, and reduce systemic toxicity [4,5]. A nanoparticle-centric approach therefore provides a clearer framework for assessing the diagnostic and therapeutic value of each platform, as well as its limitations, safety considerations, and current status in preclinical or clinical development. In the following subsections, the major nanotechnology-based systems investigated in ovarian cancer are examined individually, with emphasis on their mechanisms of action, applications, advantages, and translational challenges (Figure 1).

### 2.1. Gold Nanoparticles (AuNPs)

Gold nanoparticles (AuNPs) represent one of the most extensively studied nanotechnology platforms in ovarian cancer due to their unique optical properties, biocompatibility, and ease of surface functionalization. Their strong surface plasmon resonance enables enhanced signal amplification in biosensing applications, while their tunable size and geometry facilitate selective accumulation in tumor tissues. Functionalized AuNPs, particularly those conjugated with tumor-specific ligands such as folic acid or RGD peptides, demonstrate a high affinity for ovarian cancer cells that overexpress corresponding receptors, thereby improving both diagnostic precision and therapeutic targeting [21]. The selective behavior of AuNPs has been highlighted in multiple studies, including the review by Song et al., which emphasized their capacity to enhance tumor visualization across imaging modalities such as computed tomography and photoacoustic imaging [19].

In early detection, AuNPs have shown significant potential for improving the sensitivity of biomarker identification. Their ability to enhance optical signals has been exploited in colorimetric and plasmonic biosensors designed to detect low-abundance ovarian cancer biomarkers, including CA-125 and platelet-derived growth factor (PDGF). AuNP-aptamer conjugates targeting CA-125 have achieved detection limits in the nanogram-per-milliliter range, demonstrating superior analytical performance compared with conventional assays [12]. The sensitivity of these platforms is strongly influenced by surface modifications, with studies showing that aptamer density and orientation on the AuNP surface significantly affect binding efficiency and signal output [22]. Through these mechanisms, AuNP-based biosensors offer rapid, specific, and minimally invasive diagnostic tools that could support earlier clinical intervention.

AuNPs have also been investigated as carriers for chemotherapeutic agents and nucleic acids, leveraging their surface chemistry to enable controlled drug release and targeted delivery. Their ability to accumulate preferentially in tumor tissues enhances intratumoral drug concentration while reducing systemic exposure. In ovarian cancer models, AuNPs functionalized with folic acid have demonstrated increased uptake by folate receptor-positive cells, resulting in improved cytotoxicity of conjugated therapeutic agents. Additionally, AuNPs can serve as platforms for siRNA delivery, enabling gene silencing strategies aimed at overcoming chemoresistance. Their stability and modularity make them suitable for co-delivery of multiple therapeutic payloads, supporting combination approaches that target both cancer cells and the tumor microenvironment [24].

The dual diagnostic and therapeutic potential of AuNPs have positioned them as promising theranostic agents. Their optical properties allow real-time imaging of nanoparticle distribution, while their surfaces can be engineered to carry chemotherapeutics, siRNA, or photothermal agents. In photothermal therapy, AuNPs convert absorbed light into localized heat, inducing tumor cell apoptosis and enhancing the efficacy of chemotherapy. This multimodal functionality enables integrated treatment strategies that combine imaging, targeted delivery, and localized tumor ablation within a single platform [24,25].

Despite their advantages, AuNPs present several limitations. Tissue penetration depth remains restricted due to the optical properties of gold, which limits the effectiveness of certain imaging modalities in deep-seated tumors. Potential toxicity is another concern, particularly at high concentrations or with prolonged exposure. Although generally considered biocompatible, AuNPs may induce oxidative stress, inflammatory responses, or accumulation in organs such as the liver and spleen. Their long-term biodistribution and clearance profiles require further investigation to ensure clinical safety [25,26].

Most AuNP-based systems for ovarian cancer remain in the preclinical stage, with ongoing studies evaluating their diagnostic accuracy, therapeutic efficacy, and safety profiles. Experimental models have demonstrated that AuNPs accumulate in tumor sites at significantly higher levels than in normal tissues, supporting their potential for early detection and targeted therapy [21]. While clinical translation is still limited, the growing body of evidence underscores the promise of AuNPs as versatile platforms capable of advancing precision oncology in ovarian cancer.

### 2.2. Quantum Dots (QDs)

Quantum dots (QDs) are semiconductor nanocrystals characterized by exceptional photoluminescence, high quantum yield, and remarkable photostability, properties that make them highly attractive for diagnostic applications in ovarian cancer. Their emission spectra can be precisely tuned by adjusting particle size and composition, enabling multiplexed detection of several biomarkers within a single assay. These features have positioned QDs as powerful tools for fluorescence-based imaging and biosensing platforms designed to improve early detection and molecular characterization of ovarian tumors [27].

The strong and stable fluorescence of QDs allows for high-resolution visualization of tumor tissues and circulating biomarkers. When conjugated with antibodies or aptamers targeting ovarian cancer markers such as CA-125 and HE4, QDs enable sensitive detection in both tissue samples and body fluids. Their resistance to photobleaching provides a significant advantage over traditional organic dyes, allowing prolonged imaging sessions without signal degradation. Studies have demonstrated that QD-based probes can identify tumor-associated antigens with superior sensitivity, supporting their potential use in intraoperative imaging and real-time tumor delineation [27,28].

One of the most valuable attributes of QDs is their capacity for multiplexed detection, which is essential in ovarian cancer given the limited specificity of single biomarkers. Platforms integrating QDs with microfluidic systems have achieved simultaneous quantification of multiple circulating markers, including CA-125, HE4, and other emerging biomolecules, thereby enhancing diagnostic accuracy [29,30]. Their tunable emission wavelengths allow several QD populations to be excited by a single light source, producing distinct spectral signatures for each biomarker. This capability supports the development of advanced liquid biopsy tools capable of detecting low-abundance analytes with high precision [31].

The multifunctionality of QDs, including customizable surface chemistry and compatibility with diverse bioconjugation strategies, enables their integration into a wide range of diagnostic platforms. Their high signal-to-noise ratio and stability under physiological conditions further enhance analytical performance. However, despite these advantages, QDs face limitations related to their complex synthesis, potential aggregation in biological environments, and challenges in achieving consistent surface functionalization. Additionally, their relatively large hydrodynamic size compared with small-molecule fluorophores may affect biodistribution and cellular uptake in vivo [32].

A major barrier to clinical translation is the potential toxicity associated with QDs, particularly those containing heavy metals such as cadmium, selenium, or tellurium. Degradation of QD coatings may release toxic ions capable of inducing oxidative stress, inflammation, or DNA damage. Although surface passivation strategies and biocompatible coatings have been developed to mitigate these risks, long-term safety profiles remain insufficiently characterized. Phototoxicity during in vivo imaging also represents a concern, especially under high-intensity illumination. Consequently, most QD-based systems remain in the preclinical stage, with ongoing research focused on improving biocompatibility and reducing toxicity without compromising optical performance [31].

### 2.3. Superparamagnetic Iron Oxide Nanoparticles (SPIONs)

Superparamagnetic iron oxide nanoparticles (SPIONs) have emerged as highly promising nanomaterials for diagnostic and therapeutic applications in ovarian cancer, owing to their unique magnetic properties, biocompatibility, and capacity for surface functionalization. Their superparamagnetic behavior enables strong magnetization under an external magnetic field while preventing residual magnetism once the field is removed, a feature that enhances their safety profile and suitability for biomedical use. In ovarian cancer research, SPIONs have been primarily explored for magnetic resonance imaging (MRI) enhancement, magnetic targeting, and hyperthermia-based therapeutic strategies [33].

SPIONs significantly improve MRI contrast by altering the relaxation times of surrounding protons, thereby enabling more precise visualization of tumor tissues. Functionalization with tumor-specific ligands enhances their selective accumulation in ovarian cancer cells. For example, folic acid-conjugated Fe_3_O_4_ nanoparticles (~9.2 nm) have demonstrated preferential uptake in folate receptor-α-overexpressing ovarian cancer cells, resulting in improved tumor localization in vivo [34]. The integration of SPIONs with advanced imaging techniques such as Scanning Probe Magnetic Relaxometry (SPMR) has further increased detection sensitivity. Studies have shown that folate-targeted SPIONs combined with SPMR can identify microscopic peritoneal lesions that conventional MRI fails to detect, underscoring their potential for early detection and intraoperative guidance [35]. The high-resolution imaging enabled by SPIONs is attributed to their strong magnetic moment, which enhances signal contrast even at low concentrations [36].

Beyond imaging, SPIONs can be directed toward tumor sites using external magnetic fields, improving intratumoral accumulation and reducing systemic distribution. This magnetic targeting strategy enhances the precision of drug delivery when SPIONs are used as carriers for chemotherapeutic agents or nucleic acids. By concentrating therapeutic payloads within the tumor microenvironment, SPION-based systems may increase treatment efficacy while minimizing off-target toxicity. Their ability to respond dynamically to magnetic gradients provides a level of spatial control that is difficult to achieve with conventional drug delivery systems [35].

SPIONs also play a significant role in magnetic hyperthermia, a therapeutic approach in which nanoparticles generate localized heat upon exposure to an alternating magnetic field. This controlled heating induces apoptosis in cancer cells and can sensitize tumors to chemotherapy or radiotherapy. The combination of hyperthermia with targeted drug delivery offers a multimodal strategy capable of overcoming chemoresistance and enhancing therapeutic outcomes. In ovarian cancer models, SPION-mediated hyperthermia has shown potential to disrupt the tumor microenvironment and improve drug penetration, supporting its integration into future treatment protocols [37].

Despite their advantages, SPIONs present several safety considerations. Aggregation in biological fluids may alter their biodistribution and reduce targeting efficiency, while excessive iron accumulation can induce oxidative stress or interfere with cellular metabolism. Although SPIONs are generally considered biocompatible, their long-term clearance and potential organ deposition—particularly in the liver and spleen—require further investigation [26]. The development of SPIONs capable of generating positive contrast in low-field MRI represents a significant advancement, yet their clinical translation depends on rigorous evaluation of toxicity, stability, and immune interactions [38].

Overall, SPIONs offer a versatile platform for enhancing diagnostic accuracy and enabling targeted therapeutic interventions in ovarian cancer. Their integration into multimodal imaging and treatment strategies positions them as valuable candidates for future clinical applications, provided that ongoing research continues to address safety and translational challenges.

### 2.4. Carbon-Based Nanomaterials

Carbon-based nanomaterials, particularly carbon nanotubes (CNTs) and graphene oxide (GO), have become essential platforms in the development of biosensing and molecular detection systems for ovarian cancer. Their exceptionally large surface area, excellent electrical conductivity, and versatile surface chemistry enable the detection of biomarkers at very low concentrations, a critical requirement for early diagnosis. CNTs and GO have demonstrated high analytical performance in identifying microRNAs and tumor-associated antigens relevant to ovarian cancer, contributing to the advancement of sensitive and reproducible electrochemical biosensing technologies [39,40].

CNTs exhibit high electrical conductivity and a large specific surface area, facilitating efficient electron transfer and signal amplification in biosensors. Their integration with other nanomaterials, such as graphene oxide or metallic nanoparticles, has significantly enhanced analytical performance. For example, the combination of CNTs with chemically reduced graphene oxide (CR-GO) has demonstrated superior electrocatalytic activity due to efficient electron transfer and improved biocompatibility, enabling the detection of microRNA biomarkers at very low concentrations [41,42]. These properties position CNTs as promising platforms for biosensor development aimed at early ovarian cancer detection.

Graphene oxide (GO) is another carbon-based nanomaterial with extensive applications in biosensing, owing to its large surface area, chemical stability, and strong interactions with nucleic acids. GO functionalized with DNA probes has been used to detect microRNAs from the miR-200 family, which are known to be dysregulated in ovarian cancer. Studies have shown that GO-based biosensors can detect these microRNAs in serum samples, providing a non-invasive and highly sensitive approach for early diagnosis [39,43]. This strategy is particularly relevant given the growing interest in circulating microRNAs as emerging biomarkers for tumor monitoring.

The integration of carbon-based nanomaterials into electrochemical platforms has led to biosensors with high sensitivity, selectivity, and reproducibility. The interplay between CNTs, GO, and electrochemical detection mechanisms enables signal amplification and lowers detection limits for biomarkers such as microRNAs, CA-125, and other emerging molecular indicators [43,44]. These systems can operate effectively in complex biological matrices, including serum, due to their stability and specificity, making them strong candidates for clinical translation. Furthermore, CNT–GO hybrid systems have demonstrated superior performance in microRNA detection, benefiting from the synergistic combination of CNT conductivity and GO adsorption properties [38,39]. The theranostic versatility of carbon-based nanomaterials is further supported by recent evidence on carbon dots and their variants, which exhibit strong fluorescence, high biocompatibility, and multifunctional behavior suitable for simultaneous imaging and therapy, as highlighted by Ray et al. (2022) [45].

Although carbon-based nanomaterials offer significant advantages, they also raise concerns regarding biocompatibility and potential cytotoxicity. CNTs may induce oxidative stress, inflammation, or adverse cellular responses depending on their size, degree of functionalization, and purity. GO can interact with cellular membranes and affect membrane integrity, while its accumulation in tissues requires further investigation to determine long-term safety. Surface functionalization can substantially reduce toxicity, yet additional preclinical studies remain necessary to fully characterize the safety profiles of these nanomaterials before clinical application.

CNTs and GO represent promising platforms for developing biosensors aimed at the early detection of ovarian cancer, offering high sensitivity and the possibility of non-invasive molecular diagnostics. However, rigorous evaluation of their biocompatibility and potential toxic effects remains essential for successful clinical translation.

### 2.5. Polymeric and Liposomal Nanoparticles

Polymeric and liposomal nanoparticles constitute some of the most extensively explored nanotechnology-based systems in ovarian cancer, owing to their biocompatibility, controlled drug-release profiles, and capacity for surface functionalization, concepts that align with the foundational principles of polymer therapeutics described by Haag and Kratz (2006) [46]. Among polymeric carriers, PLGA nanoparticles have attracted considerable attention due to their biodegradability, stability, and ability to encapsulate both hydrophilic and hydrophobic therapeutic agents. Their tunable physicochemical properties allow precise modulation of drug loading, release kinetics, and targeting efficiency, making them suitable for delivering chemotherapeutics, nucleic acids, and combination therapies in ovarian cancer models [45,47]. Recent developments in folate-guided nanoplatforms further support the relevance of ligand-mediated active targeting in ovarian cancer, as demonstrated by Koti et al. (2024), who reported a core-tunable dendritic polymer capable of simultaneous imaging and drug delivery, with enhanced selectivity toward folate-receptor–positive tumor cells [48].

PLGA nanoparticles have been widely investigated for improving the delivery of paclitaxel, cisplatin, and various molecular therapeutics. Their ability to protect encapsulated agents from premature degradation enhances bioavailability and supports sustained release within the tumor microenvironment. Functionalization with ligands such as folic acid or RGD peptides increases selective uptake by ovarian cancer cells overexpressing corresponding receptors, thereby improving therapeutic efficacy while reducing systemic exposure. Studies have shown that PLGA-based formulations can modulate drug resistance pathways and enhance intracellular drug accumulation, offering a promising strategy for overcoming chemoresistance [49].

Liposomal nanoparticles represent another major class of delivery systems with established clinical relevance. Their phospholipid bilayer structure enables efficient encapsulation of chemotherapeutic agents, while PEGylation prolongs circulation time and reduces clearance by the mononuclear phagocyte system. Liposomes have demonstrated improved pharmacokinetics and reduced toxicity profiles compared with free drugs, supporting their integration into ovarian cancer therapy. Their versatility allows co-delivery of multiple agents, including siRNA and small-molecule drugs, facilitating synergistic therapeutic effects [50].

Among liposomal formulations, Doxil (pegylated liposomal doxorubicin) remains the most clinically advanced nanomedicine used in ovarian cancer. Its encapsulation strategy minimizes cardiotoxicity and enhances tumor accumulation through the enhanced permeability and retention (EPR) effect. Doxil has shown significant benefits in recurrent ovarian cancer, particularly in platinum-resistant disease, where it improves progression-free survival and reduces adverse effects compared with conventional doxorubicin [51]. Even so, challenges such as hand-foot syndrome and infusion-related reactions persist, underscoring the need for further optimization.

Paclitaxel-loaded nanoparticles, including polymeric micelles and liposomal formulations, have been developed to address solubility limitations and reduce the toxicity associated with Cremophor EL-based formulations. These nanoparticle systems enhance intratumoral drug delivery and exhibit improved tolerability. Preclinical studies indicate that targeted paclitaxel nanoparticles can increase cytotoxicity in ovarian cancer cells while limiting off-target effects, supporting their potential for clinical translation [52].

Nanoparticle-based drug delivery systems have progressed at varying rates toward clinical application. Doxil remains the most successful example, while other formulations continue to advance through preclinical and early clinical evaluation. Translational progress depends on optimizing stability, reproducibility, and large-scale manufacturing, as well as addressing regulatory considerations related to long-term safety. Taken together, polymeric and liposomal nanoparticles offer a versatile and clinically relevant platform for improving therapeutic precision in ovarian cancer, with ongoing research focused on enhancing targeting specificity, reducing toxicity, and integrating combination therapies [53].

### 2.6. microRNA and siRNA-Based Nanotechnologies

Nucleic acid–based nanotechnologies have gained considerable attention in ovarian cancer research, particularly through the development of delivery systems designed to transport microRNA (miRNA) and small interfering RNA (siRNA) molecules with high precision. These approaches aim to modulate gene expression pathways that drive tumor progression, metastasis, and chemoresistance. The therapeutic relevance of miRNAs such as the miR-200 family or oncogenic regulators including MDR1, BCL2 and TWIST has positioned RNA-based nanocarriers as promising tools for restoring chemosensitivity and inhibiting malignant phenotypes in ovarian cancer cells [52,53].

Nanoparticle-mediated gene delivery offers several advantages over conventional transfection methods, particularly by protecting RNA molecules from enzymatic degradation and enhancing their intracellular uptake. Polymeric nanoparticles, lipid-based carriers, and hybrid nanostructures have been engineered to encapsulate miRNA or siRNA sequences while ensuring controlled release and selective accumulation within tumor tissues. These systems can be functionalized with ligands such as folic acid or RGD peptides to exploit receptor overexpression on ovarian cancer cells, thereby improving delivery efficiency and minimizing off-target interactions. Through these mechanisms, RNA-loaded nanoparticles have demonstrated the capacity to silence genes associated with drug efflux, apoptosis resistance, and epithelial–mesenchymal transition, ultimately contributing to the reversal of chemoresistance [54].

One of the most extensively studied strategies involves siRNA targeting MDR1, a gene encoding P-glycoprotein, whose overexpression is strongly linked to platinum and taxane resistance. Nanoparticle-delivered siMDR1 has been shown to reduce drug efflux and enhance intracellular accumulation of chemotherapeutic agents, thereby restoring cytotoxic responses in resistant ovarian cancer cells. Similar approaches have been applied to BCL2, a key anti-apoptotic regulator, where siRNA-mediated silencing promotes apoptosis and sensitizes tumors to chemotherapy. TWIST, a transcription factor involved in epithelial–mesenchymal transition and metastatic dissemination, has also emerged as a compelling target; its inhibition through nanoparticle-delivered siRNA reduces invasiveness and may limit peritoneal spread [55].

miRNA-based nanotechnologies complement these strategies by restoring tumor-suppressive microRNAs that are frequently downregulated in ovarian cancer. Members of the miR-200 family, for instance, play a central role in maintaining epithelial identity and suppressing EMT. Their reintroduction via nanoparticle carriers has been associated with reduced metastatic potential and improved responsiveness to chemotherapy. Circulating miRNAs also hold diagnostic value, and nanoparticle-based detection systems have been developed to quantify their levels with high sensitivity, supporting both therapeutic and diagnostic applications within the same technological framework [56].

Even with these advances, several translational barriers remain. Efficient endosomal escape, long-term stability of RNA cargo, and avoidance of immune activation continue to pose challenges for clinical implementation. Furthermore, large-scale manufacturing and regulatory standardization require refinement before RNA-based nanomedicines can progress toward routine clinical use. Taken together, miRNA and siRNA nanotechnologies represent a rapidly evolving field with significant potential to reshape ovarian cancer therapy by enabling precise gene modulation, overcoming chemoresistance, and supporting the development of personalized treatment strategies [56] (Table 1).

In conclusion, the integration of nanotechnology into ovarian cancer research marks a decisive shift toward more precise, adaptable, and patient-centered therapeutic strategies. As diagnostic platforms, targeted delivery systems, and gene-modulating nanoparticle-based delivery systems continue to evolve, they collectively signal a transition from conventional cytotoxic approaches to interventions capable of addressing the molecular complexity of the disease. Although significant challenges remain—ranging from biological barriers and manufacturing constraints to long-term safety considerations—the trajectory of current evidence suggests that these obstacles can be progressively overcome through sustained interdisciplinary collaboration. Nanoparticle-based innovations therefore hold the potential not only to enhance clinical outcomes, but also to meaningfully improve the quality of life of patients by reducing toxicity, extending treatment-free intervals, and supporting a more personalized model of care.

## 3. Clinical Translation and Current Clinical Trials

Nanoparticle-based therapies have progressed from experimental concepts to clinically relevant strategies in ovarian cancer, supported by extensive preclinical evidence and an increasing number of clinical trials. Conventional chemotherapeutic agents often exhibit poor tumor specificity, resulting in systemic toxicity and insufficient intratumoral drug concentrations—limitations that contribute to treatment resistance and reduced therapeutic efficacy [59,60]. Nanoparticle delivery systems address these shortcomings by enabling controlled release, improved pharmacokinetics, and selective accumulation within the tumor microenvironment, thereby enhancing therapeutic precision while reducing adverse effects [61,62].

A major step toward clinical translation is the development of active tumor-targeting strategies. Nanoparticles functionalized with ligands such as antibodies, peptides, or hyaluronic acid exploit the overexpression of receptors including folate receptor-α and CD44 on ovarian cancer cells, improving selective uptake and minimizing off-target exposure [62,63]. Preclinical studies have shown that paclitaxel-loaded polymeric nanoparticles significantly enhance therapeutic efficacy and reduce systemic toxicity compared with conventional formulations, supporting their potential for clinical application [63,64]. These targeted systems not only improve drug accumulation in malignant tissues but also contribute to delaying recurrence, a persistent challenge in ovarian cancer management.

Nanoparticles also offer a means to overcome chemoresistance, one of the most significant barriers to successful treatment [59,65]. By co-delivering multiple therapeutic agents within a single carrier, nanoparticles can generate synergistic effects that bypass established resistance pathways. Their sustained and controlled release properties maintain therapeutic drug levels within tumors, preventing the rapid decline in concentration that often facilitates resistance development [59,60]. Furthermore, the delivery of nucleic acids such as siRNA and mRNA introduces a molecular-level therapeutic strategy capable of silencing oncogenes and reactivating tumor suppressor pathways, aligning with the broader movement toward personalized oncology [66,67].

Combination therapies incorporating nanoparticles have also advanced toward clinical evaluation. Co-encapsulation of chemotherapeutic agents with immunomodulators, including checkpoint inhibitors, enables simultaneous targeting of cancer cells and modulation of the immune response [65,68]. Nanoparticles are additionally being integrated with physical treatment modalities: magnetic nanoparticles can induce localized hyperthermia under alternating magnetic fields, while gold nanoparticles enhance photothermal therapy, both approaches amplifying the cytotoxic effects of chemotherapy and reducing recurrence risk [69,70].

Several nanoparticle platforms have progressed into clinical trials. Polymeric nanoparticles such as PLGA have gained attention for their biodegradability and controlled release characteristics [68,71], while liposomal systems—most notably Doxil (pegylated liposomal doxorubicin)—represent the most clinically advanced nanomedicine in ovarian cancer. Liposomal doxorubicin reduces cardiotoxicity and improves drug retention within tumors through the enhanced permeability and retention effect, offering a safer alternative to conventional doxorubicin [71]. Clinical studies have demonstrated its therapeutic value: in platinum- and paclitaxel-resistant ovarian cancer, Doxil achieved an overall response rate of 25.7%, including complete and partial responses, with a median progression-free survival of 5.7 months and overall survival of 11 months [69]. A larger study confirmed these findings, reporting a 16.9% response rate and a median time to progression of 19.3 weeks. The improved safety profile—marked by reduced cardiotoxicity and manageable adverse effects such as hand-foot syndrome—further supports its clinical relevance [72,73,74,75,76].

Paclitaxel-based nanoparticle formulations have also advanced into clinical testing. Nanotax, a nanoparticulate paclitaxel administered intraperitoneally, demonstrated safety and preliminary efficacy in a phase I dose-escalation study involving patients with refractory malignancies [76]. The phase III ROSELLA trial further evaluated nab-paclitaxel combined with relacorilant in platinum-resistant ovarian cancer, achieving a significant improvement in progression-free survival (6.54 vs. 5.52 months) and extending overall survival from 11.5 to 15.97 months, corresponding to a 30% reduction in the risk of progression or death [77]. These findings underscore the therapeutic potential of nanoparticle-based paclitaxel formulations, which avoid solvent-related toxicities and enable higher dosing without inducing severe allergic reactions [78,79].

Nanoparticles are also being translated into clinical strategies for gene therapy. Platforms such as polyamidoamine dendrimers and mesoporous silica nanoparticles have been used to deliver siRNA targeting genes implicated in metastasis, chemoresistance, and tumor survival. siRNA directed against TWIST achieved sustained gene knockdown and sensitized ovarian cancer cells to cisplatin in preclinical models [80,81], while mesoporous silica nanoparticles co-loaded with siRNA and cisplatin produced greater tumor reduction than cisplatin alone [80,82]. Additional studies using degradable heparin-polyethyleneimine nanoparticles to deliver survivin T34A demonstrated significant inhibition of tumor growth, reduced ascites, and increased apoptosis without notable toxicity [83,84]. These results highlight the promise of genetic nanotherapies as emerging clinical candidates capable of addressing molecular drivers of treatment failure.

Taken together, current clinical and preclinical evidence demonstrates that nanoparticle-based therapies are transitioning from experimental concepts to clinically meaningful interventions. Liposomal doxorubicin and nanoparticle paclitaxel formulations have already established therapeutic value, while gene-directed nanocarriers and multifunctional platforms represent the next frontier in personalized ovarian cancer treatment. As ongoing trials continue to refine these technologies, nanoparticle-based systems are poised to reshape therapeutic strategies by enhancing efficacy, reducing toxicity, and addressing the persistent challenge of chemoresistance.

## 4. Nanoparticles in Targeted Drug Delivery

**Figure 2:**
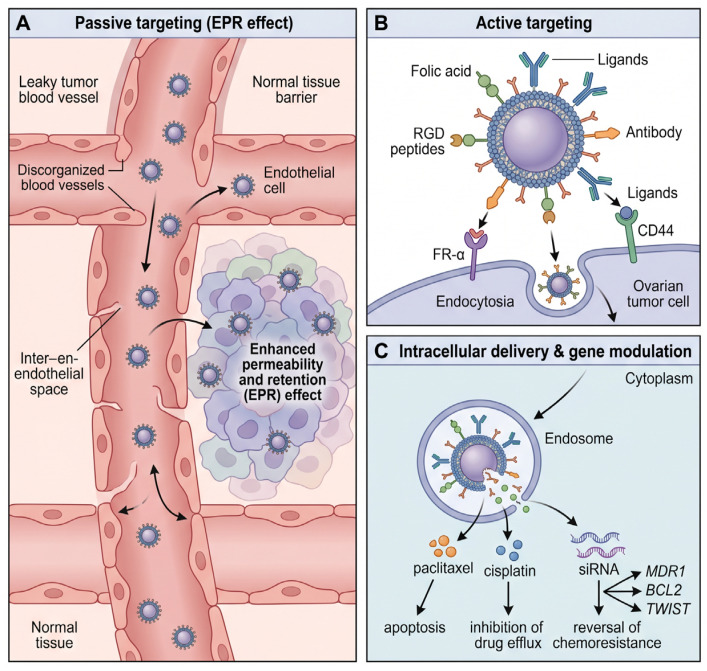
Mechanisms of Nanoparticle-Mediated Targeted Drug Delivery in Ovarian Cancer. (**A**) Passive targeting through the enhanced permeability and retention (EPR) effect enables nanoparticles to accumulate within tumor tissues. (**B**) Active targeting is achieved by functionalizing nanoparticles with ligands such as folic acid, RGD peptides, or antibodies that bind overexpressed receptors (FR-α, CD44). (**C**) Once internalized, nanoparticles release chemotherapeutic agents or gene-modulating payloads (e.g., siRNA against MDR1, BCL2, TWIST), enhancing cytotoxicity and overcoming chemoresistance.

Nanoparticle-based therapies hold significant promise for reshaping ovarian cancer management, yet their clinical translation remains constrained by several interconnected challenges that span biological, technological, regulatory, and safety-related domains. Although preclinical and early clinical studies demonstrate improved drug delivery, reduced systemic toxicity, and the capacity to overcome chemoresistance, the transition from laboratory innovation to widespread clinical adoption requires addressing these limitations in a systematic and evidence-driven manner. The main mechanism underlying nanoparticle-mediated targeted drug delivery are illustrated in Figure 2.

A major obstacle arises from the biological barriers that influence nanoparticle biodistribution, tumor penetration, and therapeutic performance. Once introduced into the bloodstream, nanoparticles interact with circulating proteins through opsonization, a process that accelerates their clearance by the mononuclear phagocyte system and diverts a substantial proportion of the injected dose to the liver and spleen [85,86]. This off-target accumulation reduces therapeutic efficacy and may increase systemic toxicity, with potential implications for fatigue, organ dysfunction, and overall health-related quality of life. Even when nanoparticles reach the tumor, they encounter additional barriers, including high interstitial pressure, dense stromal architecture, and heterogeneous receptor expression—such as variable levels of folate receptor-α or CD44—which limit their penetration and uptake [72,87,88,89]. Physiological factors unrelated to tumor biology also influence biodistribution; for example, certain nanoparticles may accumulate preferentially in ovarian tissue during ovulation, raising concerns regarding reproductive toxicity and the need to optimize dosing schedules [90]. These biological constraints highlight the importance of advanced engineering strategies capable of improving tumor selectivity, enhancing intratumoral penetration, and minimizing unintended tissue exposure.

Manufacturing and scalability represent another critical challenge. Nanoparticle production requires precise control over physicochemical parameters such as size, polydispersity, surface charge, and ligand density, all of which influence biodistribution, stability, and therapeutic efficacy [20,91]. While these parameters can be tightly regulated in laboratory settings, scaling up production for clinical trials or commercial distribution introduces variability that may compromise reproducibility and patient safety. High material costs, stringent sterility requirements, and complex purification processes further complicate large-scale manufacturing. Ensuring batch-to-batch consistency is essential, as even minor deviations can alter therapeutic performance or toxicity profiles. These manufacturing constraints also have socioeconomic implications, particularly in resource-limited settings where financial barriers already restrict access to innovative therapies [90]. Addressing these challenges requires the development of standardized, cost-effective, and scalable production platforms capable of supporting widespread clinical adoption.

Regulatory and commercial complexities further impede the translation of nanomedicine into clinical practice. Nanoparticles often incorporate multiple functional components—drug payloads, targeting ligands, polymers, or inorganic cores making classification difficult within existing regulatory frameworks [92]. Their high surface-to-volume ratio can lead to unpredictable biological interactions, and early safety testing may not detect rare or delayed adverse events that emerge only in larger populations. The substantial financial investment required for research, development, and regulatory approval—often exceeding one billion dollars poses additional commercial risks, potentially discouraging industry engagement [93,94]. These challenges underscore the need for early collaboration between developers and regulatory agencies, as well as the integration of patient-reported outcomes to ensure that therapeutic advances translate into meaningful improvements in daily functioning and quality of life.

Long-term toxicity remains one of the most important unresolved issues in nanomedicine. While many nanoparticle systems demonstrate favorable short-term safety profiles, their long-term biodistribution, degradation, and clearance are not fully understood. Persistent accumulation in organs such as the liver or spleen may contribute to chronic inflammation or organ dysfunction, with potential consequences for physical well-being and functional independence [95]. Gene-delivery platforms introduce additional concerns, as repeated administration of siRNA or plasmid-based therapies may have cumulative effects that are not yet fully characterized. Even when preclinical studies report minimal toxicity—such as the use of heparin-polyethyleneimine nanoparticles delivering survivin T34A, which significantly reduced tumor burden without notable adverse effects [83,84]—long-term human data remain essential to confirm safety across diverse patient populations. The development of biodegradable and biocompatible materials, including PLGA-based systems, represents a promising strategy for mitigating long-term risks while supporting scalable production and combination therapy approaches [47].

Looking forward, the future of nanoparticle-based therapy in ovarian cancer will depend on addressing these interconnected challenges through advances in materials science, tumor biology, immunology, and manufacturing technology. Continued refinement of targeting strategies, improved understanding of nanoparticle cell interactions, and the development of standardized regulatory pathways will be essential for ensuring safe and equitable clinical translation. As these innovations progress, nanomedicine holds the potential to reshape ovarian cancer management by enhancing therapeutic precision, reducing toxicity, and supporting more personalized and patient-centered care (Table 2).

## 5. Conclusions

Over the past two decades, nanoparticle-based technologies have progressed from theoretical constructs to clinically validated tools capable of reshaping therapeutic strategies in ovarian cancer. Formulations such as liposomal doxorubicin and nanoparticle albumin-bound paclitaxel have demonstrated improved pharmacokinetic behavior, reduced systemic toxicity, and preserved antitumor efficacy, particularly in recurrent or treatment-resistant disease [76,80,87,98,99,100,101,102,103,104,105,106]. These advances extend beyond tumor control, contributing to improved treatment tolerability and supporting a more patient-centered approach to care. As the field evolves, the integration of nanotechnology into ovarian cancer management increasingly reflects a dual objective: prolonging survival while preserving functional status, minimizing cumulative toxicity, and maintaining psychosocial well-being.

Despite these promising developments, several translational challenges remain. Large-scale, reproducible manufacturing of nanoparticles, long-term biosafety evaluation, and precise control over biodistribution continue to represent critical barriers to widespread implementation [107,108,109]. Ensuring that nanoparticles do not accumulate in non-target tissues and do not induce chronic inflammatory or immunogenic responses is essential for guaranteeing both safety and sustained therapeutic benefit [107,110]. These considerations are particularly relevant in maintenance regimens and prolonged treatment courses, where cumulative exposure may influence long-term quality of life.

The field is now transitioning from improved drug delivery toward molecular-level therapeutic modulation. Emerging platforms including gene-silencing nanocarriers, stimuli-responsive intelligent release systems, and multifunctional theranostic nanoparticles—enable simultaneous diagnosis, targeted therapy, and real-time monitoring of treatment response [111]. Notably, CRISPR-loaded nanoparticle systems currently under early clinical evaluation aim to directly edit resistance-associated gene sequences within tumor cells, offering a strategy to address the biological drivers of chemoresistance and illustrating the transformative potential of gene-editing nanotherapies [81,112]. These innovations exemplify the broader movement toward precision nanomedicine, in which treatment is tailored not only to tumor biology but also to minimizing patient burden.

As nanotechnology advances, its integration into ovarian cancer care must increasingly incorporate patient-reported outcomes and health-related quality-of-life metrics as core endpoints in clinical trials. Demonstrating reductions in neuropathy, cardiotoxicity, fatigue, hospitalization rates, and treatment discontinuation will be essential to substantiate the real-world benefits of nanomedicine beyond conventional survival metrics. In this context, nanoparticle-enabled early diagnosis, targeted drug delivery, and resistance-modulating strategies may contribute not only to improved tumor control but also to prolonged treatment-free intervals, reduced therapeutic burden, and preservation of functional independence [113,114].

Ultimately, nanoparticles represent a promising frontier in ovarian cancer therapy, offering opportunities for precise drug delivery, resistance modulation, early detection, and real-time therapeutic monitoring [105,112,115,116]. Their potential to transform ovarian cancer care lies not solely in improving clinical outcomes, but in reshaping the overall patient experience—from diagnosis through long-term management. To realize this vision, coordinated collaboration among academic researchers, industry partners, and regulatory authorities is essential. Future efforts must prioritize biologically informed nanoparticle design, standardized and scalable manufacturing processes, rigorous long-term safety assessment, and equitable access to emerging nanotherapies [117,118]. Clear regulatory frameworks and multidisciplinary partnerships will be instrumental in accelerating safe and effective clinical translation.

Synthesizing these developments, nanoparticle-based strategies offer a multifaceted and increasingly refined approach to ovarian cancer management. By integrating targeted therapy, resistance modulation, and enhanced diagnostic precision, these technologies hold the potential to redefine treatment paradigms shifting from survival-driven models toward more precise, effective, and human-centered care.

## Figures and Tables

**Table 1:** Nanotechnology-based systems explored for early ovarian cancer detection, highlighting mechanisms, advantages, limitations, representative targets, and validation stages.

Nanotechnology Type	Mechanism	Advantages	Limitations	Typical Targets/Examples	Validation Stage	Ref.
Folate-modified nanoparticles	Ligand-functionalized particles bind to folate receptors overexpressed in ovarian cancer, enabling selective accumulation.	High specificity for folate receptor-positive tumors; improved diagnostic precision.	Variable receptor expression; potential immunogenicity.	Folate receptor-α on ovarian cancer cells.	Preclinical and clinical evaluation.	[45,46]
Magnetic iron oxide nanoparticles (SPIONs)	Magnetic guidance enhances tumor localization and MRI contrast.	Superior imaging contrast; controllable targeting using external magnetic fields.	Possible aggregation; dose-dependent toxicity.	Fe_3_O_4_ nanoparticles for MRI enhancement.	Experimental; promising imaging applications.	[17,24,25]
microRNA-based nanotechnology	Encapsulation of miRNAs enables targeted delivery and gene modulation in ovarian cancer cells.	Sensitive detection of disease-specific miRNA signatures.	Stability and in vivo delivery remain challenging.	miRNA profiles in patient serum samples.	Early research; clinical validation required.	[54,55,56]
Immunotherapy-linked nanocarriers	Nanocarriers deliver immune modulators to enhance antitumor immunity.	Strengthens immune responses through targeted delivery.	Risk of unintended immune activation.	Tumor-associated antigens.	Ongoing clinical trials.	[53]
Contrast-enhanced ultrasound nanoparticles	Nanoparticles improve acoustic contrast for enhanced tumor visualization.	Higher imaging resolution and improved lesion localization.	Dependent on nanoparticle pharmacokinetics; potential side effects.	Ultrasound contrast agents.	Under clinical validation.	[53,54]
Gold nanoparticles (AuNPs)	Surface-enhanced Raman scattering enables sensitive biomarker detection.	Excellent biocompatibility; strong plasmonic properties.	Limited tissue penetration; toxicity at high doses.	CA-125 and other tumor markers.	Preclinical; clinical potential under investigation.	[24,25,26,34]
Quantum dots (QDs)	Antibody-conjugated QDs enable fluorescence imaging and multiplex biomarker detection.	High sensitivity; tunable emission; robust multiplexing.	Phototoxicity and stability concerns in vivo.	CA-125, HER2, additional biomarkers.	Various stages; active research field.	[31,32]
Carbon nanotubes (CNTs)	CNTs support drug delivery, imaging, and electrochemical biosensing.	Large surface area; excellent electrical conductivity.	Biocompatibility concerns; potential cytotoxicity.	Cancer biomarkers; therapeutic payloads.	Research in progress; theranostic potential.	[39,40,41,42,43,44,57,58]

**Table 2:** Nanoparticle-based drug delivery systems in ovarian cancer and their current clinical development status.

Nanoparticle Platform	Therapeutic Payload/Mechanism	Clinical Status	Regulatory Status	Key Outcomes
Liposomal doxorubicin (Doxil/Caelyx)	Doxorubicin; passive targeting via EPR; reduced cardiotoxicity	Approved; multiple Phase II–III trials	FDA/EMA approved	Improved PFS and reduced cardiotoxicity in recurrent ovarian cancer [72,73,74,75,76,87]
Nab-paclitaxel	Paclitaxel bound to albumin nanoparticles; solvent-free formulation	Approved; Phase III (ROSELLA)	FDA approved	Improved PFS and OS when combined with relacorilant [77,78,88]
Nanotax (nanoparticulate paclitaxel)	Intraperitoneal nanoparticle paclitaxel	Phase I	Not approved	Safe intraperitoneal administration; sustained local drug levels [76]
PLGA nanoparticles	Paclitaxel, cisplatin, siRNA; controlled release; receptor-targeted delivery	Preclinical	Not approved	Enhanced intracellular accumulation; reversal of MDR pathways [47,49,96]
Mesoporous silica nanoparticles (MSNs)	siRNA (TWIST, MDR1), cisplatin; high loading capacity	Preclinical	Not approved	Significant tumor reduction in xenograft models [80,81,82]
Gold nanoparticles (AuNPs)	Drug conjugates, siRNA, photothermal therapy	Preclinical	Not approved	Enhanced tumor uptake; theranostic potential [24,25,26,97]
SPIONs	Magnetic targeting; drug delivery; hyperthermia	Preclinical	Not approved	MRI enhancement; improved intratumoral accumulation [23,24,25]
Carbon-based nanomaterials (CNTs, GO)	Drug delivery; microRNA modulation	Preclinical	Not approved	High loading capacity; biosensing potential; toxicity concerns [39,40,43,44,57,58]
siRNA/microRNA nanocarriers	Gene silencing (MDR1, BCL2, TWIST)	Preclinical	Not approved	Reversal of chemoresistance; apoptosis induction [54,55,56,80,81,82,83,84]

## Data Availability

No new data were created or analyzed in this study.
